# Supplementary material for: Adsorption of Hydrolysed Polyacrylamide onto Calcium Carbonate
Source: Polymers (Basel). 2022 Jan 20;14(3):405. doi: 10.3390/polym14030405 (PMC8838080; doi:10.3390/polym14030405)
Supplement: Supplementary file 1 [file polymers-14-00405-s001.zip › polymers-1534195-SI.pdf]

polymers

Supplementary Material

Article

Adsorption of Hydrolysed Polyacrylamide onto Calcium Carbonate

Jin Hau Lew [1], Omar K. Matar [1], Erich A. Müller [1], Myo Thant Maung Maung [2] and Paul F. Luckham [1,*]

[1] Department of Chemical Engineering, Imperial College London, London SW7 2AZ, UK; s.lew20@imperial.ac.uk (J.H.L.); o.matar@imperial.ac.uk (O.K.M.); e.muller@imperial.ac.uk (E.A.M.)
[2] PETRONAS Research Sdn. Bhd., Bandar Baru Bangi 43000, Selangor, Malaysia; maungmyothant@petronas.com
* Correspondence: p.luckham01@imperial.ac.uk

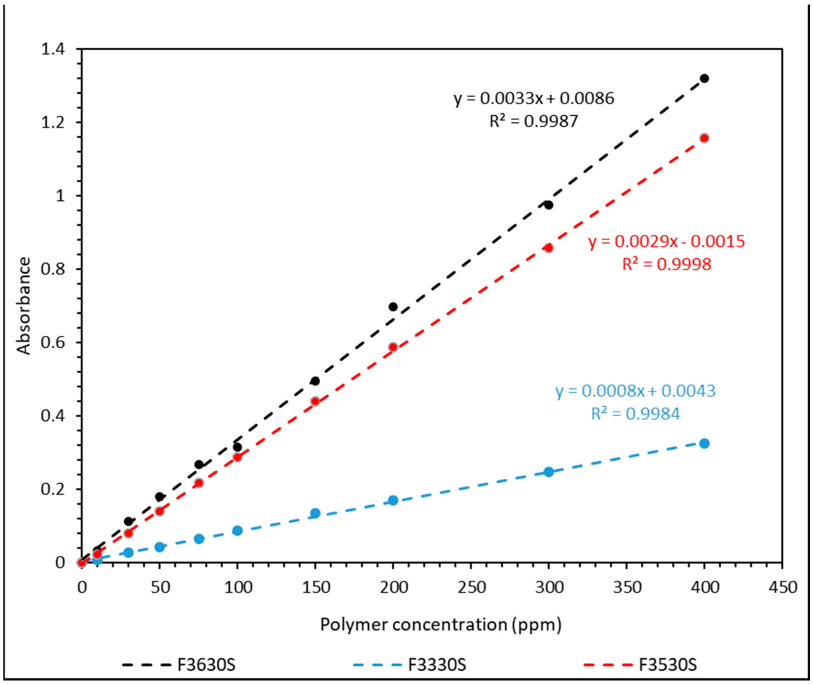

Figure S1. Calibration curve of F3330S, F3530S and F3630S.

Citation: Lew, J.H.; Matar, O.K.; Müller, E.A.; Maung, M.T.M.; Luckham, P.F. Adsorption of Hydrolysed Polyacrylamide onto Calcium Carbonate. *Polymers* **2022**, *14*, 405. 10.3390/polym14030405

Academic Editors: How Wei Benjamin Teo, Anutosh Chakraborty and Mujib. L. Palash

Received: 15 December 2021
Accepted: 17 January 2022
Published: 20 January 2022

Publisher's Note: MDPI stays neutral with regard to jurisdictional claims in published maps and institutional affiliations.

Copyright: © 2022 by the authors. Submitted for possible open access publication under the terms and conditions of the Creative Commons Attribution (CC BY) license (https://creativecommons.org/licenses/by/4.0/).

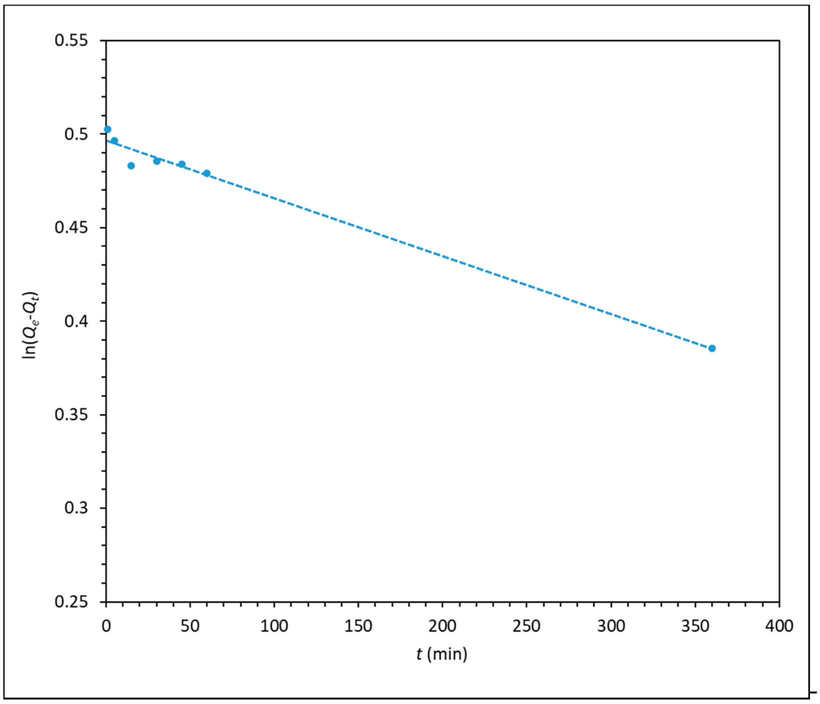

Figure S2. ln(Q_e-Q_t) versus t (pseudo-first order kinetic model) for the adsorption of 300ppm F3530S onto $CaCO_3$. (R^2 = 0.9869).

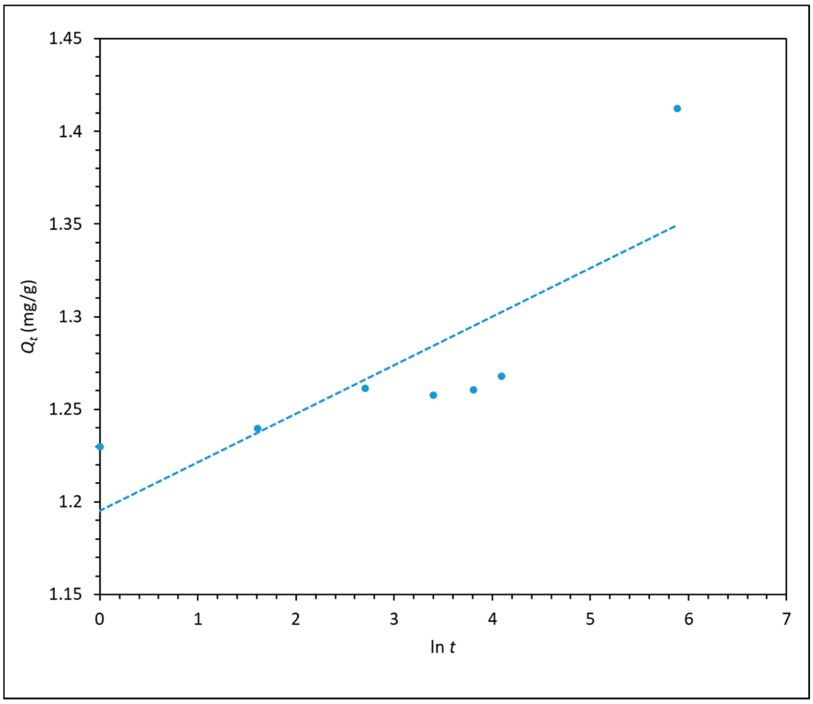

Figure S3. Q_t versus ln t (Elovich kinetic model) for the adsorption of 300ppm F3530S onto CaCO3. (R^2 = 0.6394).

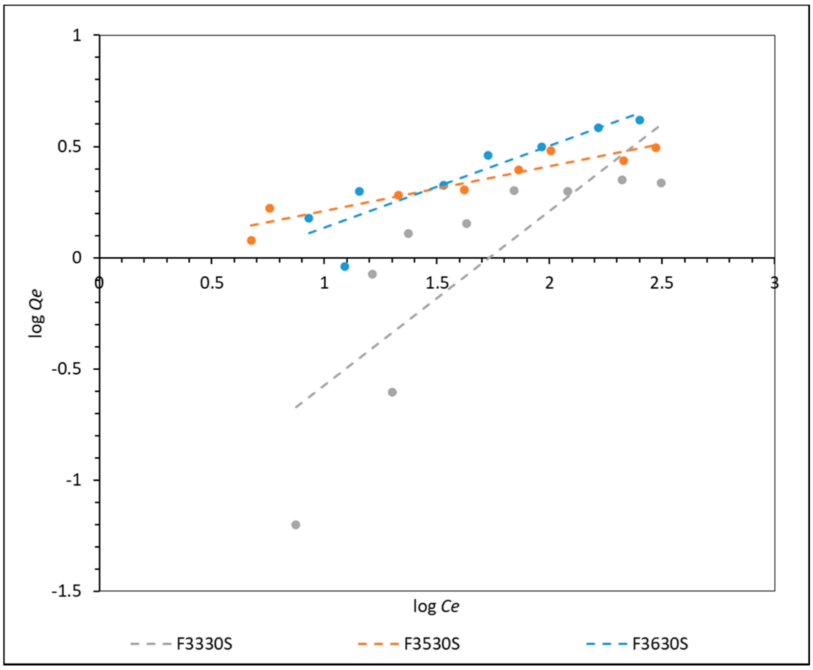

Figure S4. Freundlich adsorption isotherm model of F3330S, F3530S and F3630S adsorption onto $CaCO_3$.

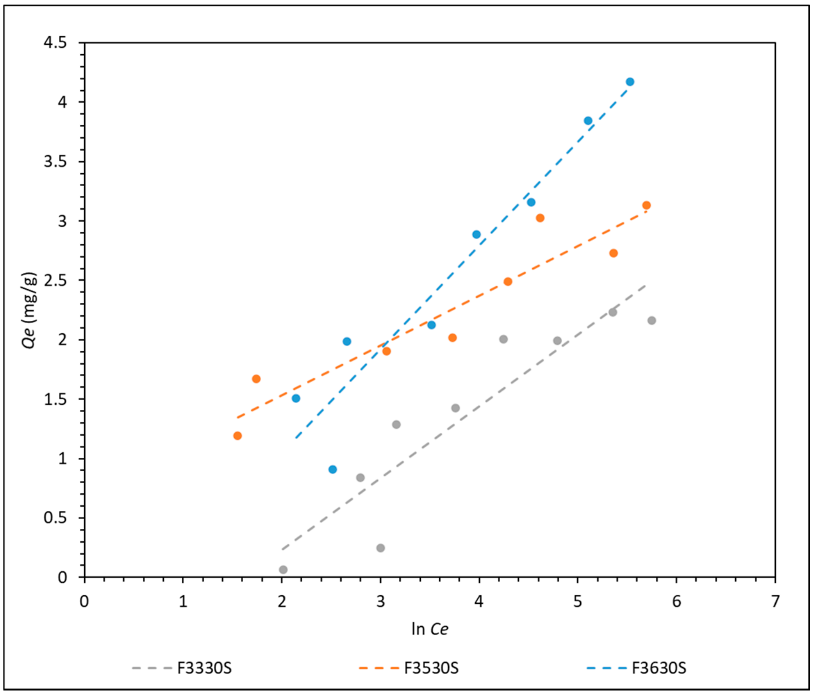

Figure S5. Temkin adsorption isotherm model of F3330S, F3530S and F3630S adsorption onto $CaCO_3$.

Table S1. Adsorbed amount of F3330S, F3530S and F3630S onto CaCO3 against polymer concentration.

**Concentration (ppm)**	10	30	50	75	100	150	200	300	400
**F3330S (mg/m²)**	0.007	0.028	0.093	0.143	0.159	0.223	0.221	0.248	0.240
**F3530S (mg/m²)**	0.090	0.133	0.185	0.211	0.224	0.276	0.336	0.303	0.348
**F3630S (mg/m²)**	0.080	0.101	0.167	0.221	0.236	0.321	0.351	0.427	0.463

Table S2. Example standard deviation and error from triplicated results of F3330S.

Concentration (ppm)	Adsorbed polymer per surface area of $CaCO_3$ (mg/m²)			Standard deviation	Standard error
	#1	#2	#3		
0	0	0	0	0.00E+00	0.00E+00
10	0.008	0.012	0.001	5.14E-03	2.97E-03
30	0.028	0.028	0.027	2.25E-04	1.30E-04
50	0.082	0.094	0.105	1.15E-02	6.62E-03
75	0.144	0.139	0.145	3.51E-03	2.03E-03
100	0.158	0.158	0.160	9.83E-04	5.67E-04
150	0.220	0.224	0.224	2.32E-03	1.34E-03
200	0.217	0.222	0.224	3.37E-03	1.94E-03
300	0.248	0.248	0.248	6.82E-05	3.94E-05
400	0.239	0.241	0.240	1.22E-03	7.06E-04

Table S3. Adsorption result of F3530S over different stirring time.

Concentration (ppm)	10	30	50	75	100	150	200	300
**1h (mg/m²)**	0.011	0.053	0.086	0.087	0.087	0.090	0.131	0.096
**6h (mg/m²)**	0.030	0.051	0.173	0.177	0.208	0.177	0.215	0.196
**18h (mg/m²)**	0.072	0.129	0.178	0.232	0.260	0.276	0.324	0.326
**24h (mg/m²)**	0.077	0.123	0.177	0.216	0.222	0.263	0.328	0.328
**72h (mg/m²)**	0.101	0.087	0.138	0.239	0.251	0.271	0.345	0.306

Table S4. Relation coefficient of adsorption model.

Polymers	Langmuir (R^2)	Freundlich (R^2)	Temkin (R^2)
F3330S	0.9955	0.646	0.8478
F3530S	0.9893	0.8888	0.8924
F3630S	0.9726	0.8149	0.9252

Table S5. Adsorption information from Langmuir Isotherm.

Polymers	Q_m (mg/m²)	K_L (L/mg)
F3330S	0.263	0.045
F3530S	0.343	0.097
F3630S	0.496	0.039

Table S6. Tabulated R_L values.

C_0 (mg/L)	R_L		
	F3330S	F3530S	F3630S
10	0.6887	0.5077	0.7208
30	0.4244	0.2558	0.4625
50	0.3067	0.1710	0.3405
75	0.2278	0.1209	0.2560
100	0.1812	0.0935	0.2052
150	0.1285	0.0643	0.1468
200	0.0996	0.0490	0.1143
300	0.0687	0.0332	0.0792
400	0.0524	0.0251	0.0606
